# Long-term outcomes after surgical repair of subvalvular aortic stenosis in pediatric patients

**DOI:** 10.3389/fcvm.2022.1033312

**Published:** 2022-12-02

**Authors:** Johanna Schlein, Felix Wollmann, Alexandra Kaider, Dominik Wiedemann, Harald Gabriel, Stephan Hornykewycz, Eva Base, Ina Michel-Behnke, Günther Laufer, Daniel Zimpfer

**Affiliations:** ^1^Department of Cardiac Surgery, Medical University of Vienna, Vienna, Austria; ^2^Center for Medical Statistics, Informatics, and Intelligent Systems, Medical University of Vienna, Vienna, Austria; ^3^Department of Internal Medicine II, Division of Cardiology, Medical University of Vienna, Vienna, Austria; ^4^Department of Anaesthesia, Intensive Care Medicine and Pain Medicine, Division of Cardiac Thoracic Vascular Anaesthesia and Intensive Care Medicine, Medical University of Vienna, Vienna, Austria; ^5^Department of Children and Adolescent Medicine, Division of Pediatric Cardiology, Medical University of Vienna, Vienna, Austria

**Keywords:** subvalvular aortic stenosis, subvalvular aortic membrane, subaortic stenosis, subaortic membrane resection, subaortic myectomy

## Abstract

**Objectives:**

Subvalvular aortic stenosis (SAS) can occur as discrete or tunnel-like obstruction of the left ventricular outflow tract and as progressive disease often leads to aortic valve regurgitation. We report our 30-year single-center experience after surgical repair of SAS.

**Methods:**

A retrospective chart review of all patients aged < 18 years, who underwent surgical repair of SAS from May 1985 to April 2020, was conducted. Mortality was cross-checked with the national health insurance database (93.8% complete mortality follow-up in April 2020). Survival and competing risks analysis were used to analyze the primary endpoints survival and incidence of reoperations.

**Results:**

From May 1985 until April 2020 103 patients (median age 5.5 years) underwent surgical repair of SAS. Survival was 90.8% at 10 years and 88.7% at 20 and 30 years. Age < 1 year at time of surgery, Shone’s complex, mitral stenosis and concomitant mitral valve surgery were associated with mortality. The cumulative incidence of reoperation for SAS was 21.6% at 10 years, 28.2% at 20 and 30 years. The incidence of reoperation for SAS did not differ between the myectomy, membrane resection and combined myectomy and membrane resection groups. The cumulative incidence of reoperation on the aortic valve was 13.5% at 20 years.

**Conclusion:**

Recurrence rate of SAS is not to be neglected, though surgical repair of subaortic stenosis has good long-term results. Patients who needed a combined membrane resection and septal myectomy are not more prone to recurrence than patients who underwent solitaire myectomy or membrane resection.

## Introduction

Subvalvular aortic stenosis (SAS) has a broad disease spectrum and a progressive disease nature. SAS can occur as a solitaire subvalvular membrane in the left ventricular outflow tract (LVOT) or a minor fibrous muscular ridge on the subvalvular ventricular septum (discrete SAS). In severe cases SAS occurs as a narrow fibromuscular tunnel-like obstruction of the LVOT (1, 2). The stenosis causes turbulent blood flow, which damages and scares the aortic valve tissue leading to aortic regurgitation due to aortic valve prolapse (3). Membranous subaortic tissue might extend onto the aortic valve leaflet, which leads to restricted valve mobility and mal-adaptation of the leaflets. Progression of valve regurgitation after subaortic resection is common (1–3), with reoperation rates for valve repair or replacement after initial subaortic resection as high as 20% (1). Long-term outcomes regarding late reoperation and reoperation on the aortic valve remain incompletely defined in pediatric patients with SAS (4). We reviewed our long-term single-center experience with SAS repair in pediatric patients to report on mortality and reoperation rates.

## Patients and methods

### Patients

A chart review of all patients less than 18 years of age at time of surgery who underwent SAS repair between May 1985 and April 2020 was conducted. The study was approved by the institutional review board of the center (Ethics committee submission number: 1414/2019) and patient consent was waived due to the retrospective study design. Patients, who underwent myectomy and/or membrane resection for SAS were included. Patients undergoing modified Konno procedure were excluded from this study. In the mentioned study period 11 pediatric patients underwent modified Konno procedure. A mortality cross-check with the national health insurance database was conducted. Mortality follow-up is available until April 2020. Six patients (6/103, 5.8%), who were transferred and followed-up at foreign countries could not be looked up in the database. In the conducted survival analysis patients, who could not be cross-checked were censored at the last cardiac follow-up at the center. Median follow-up time was 10.5 years (interquartile range [IQR], 4.9–19.8) with a longest follow-up of 34.6 years. Early mortality included patients, who died within the first 30 days after the procedure or in-hospital during the index hospitalization of the procedure. Reoperation was evaluated for SAS reoperation as well as for aortic valve reoperation (valve repair and aortic valve replacement). Modified Konno procedure and Ross-Konno procedure were analyzed as reoperation for subvalvular aortic stenosis and in case of the Ross-Konno procedure for aortic valve reoperation respectively. Cumulative incidences of SAS reoperation were compared between the following surgical cohorts: myectomy, membrane resection, combined myectomy, and subvalvular membrane resection.

### Indication for surgery and surgical techniques

Indication for surgical treatment is decided upon the peak LVOT gradient. Patients with a peak LVOT gradient of >50 mmHg should undergo surgical treatment. In patients with a peak LVOT gradient between 30 and 50 mmHg decision for surgical treatment is based the symptoms and the progression of aortic regurgitation. A subvalvular membrane might be diagnosed in the preoperative echocardiography, but the surgical approach is decided intraoperatively at inspection of the LVOT, depending on the anatomy of the stenosis concerning presence of a membrane and extent of septal hypertrophy. The extent of myectomy is based on the individual anatomy and size of the LVOT and has to be performed under precaution of the conduction system. Transaortic septal myectomy is the standard approach in pediatric patients at the center. No transmitral or apical septal myectomy was performed in pediatric patients during the mentioned study period. During surgery, the LVOT is accessed *via* an oblique aortotomy, the aortic valve is retracted, allowing for resection of the membrane and septal myectomy in the setting of myocardial hypertrophy (5). Redundant subvalvular membrane tissue which extends onto the aortic valve, mitral valve and/or accessory papillary muscles attachments is resected.

### Statistical analysis

Continuous data are expressed as mean ± standard deviation, whilst skewed continuous data are expressed as median with interquartile range (IQR). Categorical variables were shown as frequencies and percentages. The median follow-up time was estimated by the inverse Kaplan-Meier method (6). Survival probabilities were calculated by the Kaplan-Meier method. To quantify the association of factors with survival univariable Cox proportional hazards regression models were calculated. The probability of reoperation for SAS or reoperation on the aortic valve (repair and replacement) was estimated by the cumulative incidence function considering death and cardiac transplantation as competing events. Gray’s test was used to determine differences between cumulative incidence functions. To quantify the effect of the continuous prognostic factors age and year of operation on the reoperation risk, univariable Cox proportional cause-specific hazards regression models were performed. Statistical significance was set at *p* < 0.05. Data was analyzed using the software package SPSS^®^ 26 (IBM Corp., Chicago, IL, USA) and SAS 9.5 (SAS Institute Inc., Cary, NC, USA).

## Results

### Patient demographic and operative characteristics

From May 1985 until April 2020, 103 patients (58/103, 56.3% male; 24/102, 23.3% bicuspid aortic valve) underwent repair of SAS. Demographic and operative data is seen in Table 1. SAS repair was performed as following: myectomy: 29/103, 28.2%; membrane resection: 44/103, 42.7%; combined myectomy and membrane resection: 30/103, 29.1%. The median preoperative maximum instantaneous gradient across the LVOT was 62 mmHg (IQR 50–81 mmHg). The median preoperative maximum instantaneous gradient across the LVOT did not differ (*p* = 0.571) between myectomy, membrane resection and combined myectomy, and membrane resection groups with 67 mmHg (IQR 37–95 mmHg), 62 mmHg (IQR 47–78 mmHg) and 67.5 mmHg (IQR 55–84 mmHg). Median age at time of surgery was 5.5 years (IQR 1.6–10.8 years) and 43 patients (43/103, 41.7%) had already undergone previous cardiac surgery before SAS repair. Ten patients (10/103, 9.7%) had undergone previous repair of atrioventricular canal with a mean time from corrective surgery to SAS repair of 10.6 ± 3.8 years. Two patients (2/103, 1.9%) had been treated with radiofrequency ablation for SAS earlier. Four patients (4/103, 3.9%) had undergone surgery on the aortic valve (surgical aortic valvuloplasty: *n* = 3, 2.9%; surgical aortic valve reconstruction: *n* = 1, 1%). Concomitant aortic valve procedures at time of SAS repair were performed in 18 (18/103, 17.5%) cases and are listed in Table 1. Concomitant right ventricular outflow tract myectomy was necessary in 9 (9/103, 8.7%) cases.

**TABLE 1:** Demographic and operative data.

Demographic	Patients
Number	103 (100)
Male	58 (56.3)
**Diagnoses**	
Atrioventricular canal	12 (11.7)
Hypertrophic obstructive cardiomyopathy	17 (16.5)
Right ventricular outflow tract obstruction	10 (9.7)
VSD/ASD/PFO/PDA	34 (33)
Coarctation of the aorta	22 (21.4)
Hypoplastic aortic arch	12 (11.7)
Shone’s complex	10 (9.7)
Mitral valve stenosis	10 (9.7)
Borderline left ventricle	4 (3.9)
**Aortic valve anatomy**	
Bicuspid	24 (23.3)
Tricuspid	67 (65)
Quadricuspid	1 (1)
Unknown	11 (10.7)
**Previous cardiac interventions**	
Any previous cardiac intervention	49 (47.6)
Any aortic valve intervention	8 (7.8)
Any intervention for subvalvular aortic stenosis	2 (1.9)
Balloon aortic valvuloplasty	4 (3.9)
Radiofrequency ablation for subvalvular aortic stenosis	2 (1.9)
Other percutaneous intervention	7 (6.8)
Any surgical intervention	43 (41.7)
Surgical aortic valvuloplasty	3 (2.9)
Surgical valve reconstruction	1 (1)
Surgical repair of atrioventricular canal	10 (9.7)
Other surgical intervention	28 (27.2)

**Operative**	**Number**

**Surgical era**	
1985–1995	21 (20.4)
1996–2005	32 (31.1)
2006–2020	50 (48.5)
**Age at time of surgery**	
Median age at time of surgery	5.5 (1.6–10.8)
Neonates	2 (1.9)
<1 year of age (including neonates)	16 (15.5)
1–5 years of age	39 (37.9)
6–13 years of age	36 (35)
14–18 years of age	12 (11.7)
Weight at time of surgery (kg)	20 (11–34.7)
Height at time of surgery (cm)	116 (84–140)
BSA_*Haycock*_ at time of surgery	0.8 (0.5–1.2)
**Surgical repair of subvalvular stenosis**	
Myectomy	29 (28.2)
Membrane resection	44 (42.7)
Combined myectomy and membrane resection	30 (29.1)
Aortic regurgitation as indication for subvalvular surgery	37 (35.9)
Grade I	20 (19.4)
Grade II	11 (10.7)
Grade III	6 (5.8)
Concomitant aortic valve involvement	18 (17.5)
**Indication for concomitant aortic valve involvement**	
Valve stenosis	6 (5.8)
Valve regurgitation	5 (4.9)
Combined valve disease	4 (3.9)
Leaflet lesion during subvalvular resection	2 (1.9)
Concomitant right ventricular outflow tract myectomy	9 (8.7)
Concomitant VSD/ASD/PFO/PDA closure	21 (20.4)
Concomitant aortic arch surgery	4 (3.9)
Concomitant supravalvular surgery	2 (1.9)
Concomitant mitral valve intervention	10 (9.7)
Concomitant pulmonary valve intervention	3 (2.9)
Concomitant correction of coarctation of the aorta	3 (2.7)
ACCT (min)	37 (25–70)
CPB (min)	65 (50–126)
Circulatory arrest	5 (4.9)

Values are presented as *n*, *n* (%), median (interquartile range). ACCT, aortic cross clamp time; ASD, atrium septal defect; BSA, body surface area; CPB, cardiopulmonary bypass; PDA, persistent ductus arteriosus; PFO, persistent foramen ovale; VSD, ventricular septal defect.

### Early outcome

In Table 2 the short-term postoperative outcomes are seen. Early mortality was 6.8% (7/103). All early deaths occurred in patients with complex congenital heart disease or hypertrophic obstructive cardiomyopathy (HOCM). Early and late deaths are seen in Table 3. Permanent pacemaker implantation in the setting of AV-block was necessary in four patients undergoing solitaire myectomy (4/103; 3.9%; three patients with HOCM).

**TABLE 2:** Postoperative outcomes.

Characteristic	Number
Permanent pacemaker implantation	4 (3.9)
Delayed sternal closure	3 (2.9)
Revision for bleeding	1 (1)
Subxiphoidal drainage of pericardial effusion	1 (1)
Deep sternal wound infection	0 (0)
Ventilation (days)	1 (0–1)
ICU stay (days)	2 (1–3)
Hospital stay (days)	11 (8–14)
Dialysis/Hemofiltration	2 (1.9)
ECMO	3 (2.9)
Early mortality	7 (6.8)

Values are presented as *n*, *n* (%), median (interquartile range). ECMO, extracorporeal membrane oxygenation; ICU, intensive care unit.

**TABLE 3:** Early and late deaths.

Patient no. (sex)	Surgery (year)	Age at surgery	Diagnosis/Previous intervention	Reoperation (postoperative days/years)	Death (postoperative days/years)	Cause of death
**Early deaths**						
No. 1 (m)	Myectomy (2005)	8 days	DORV, Shone’s complex with borderline left ventricle		Mors in tabulam	Preoperative NEC, myocardial decompensation intraoperative
No. 2 (m)	Myectomy (1995)	1.5 months	DORV-TGA, CoA after subclavian flap and pulmonary artery banding		3 days	Myocardial decompensation on ECMO
No. 3 (f)	Membrane resection (1994)	9.5 years	Dextrocardia, bicuspid aortic valve, CoA after subclavian flap, mitral valve anomaly with mitral regurgitation, tricuspid regurgitation	Mitral valve reconstruction and De-Vega plasty of the tricuspid valve on the excised and then auto-transplanted heart (4 days)	4 days	Myocardial decompensation on ECMO
No. 4 (m)	Myectomy (1999)	1 day	Shone’s with hypoplastic left heart hypoplastic mitral valve, bicuspid aortic valve, interrupted aortic arch	Re-opening of VSD and ASD, pulmonary banding (7 days) Norwood I (12 days)	15 days	Myocardial decompensation on ECMO
No. 5 (m)	Combined myectomy and membrane resection (1999)	7.5 months	AV-canal with parachute MV and goose neck formation of LVOT, pulmonary artery banding	Mechanical mitral valve replacement (13 days)	19 days	Myocardial decompensation on ECMO
No. 6 (f)	Combined myectomy and membrane resection (1993)	9.6 years	HOCM, VSD		19 days	MOV, sepsis after laparotomy for ileus (15 days)
No. 7 (m)	Myectomy (2019)	4.4 years	HOCM, mitral valve regurgitation, bicuspid aortic valve		1.5 months	MOV
**Late deaths**						
No. 8 (m)	Myectomy (2014)	6 months	HOCM, mitral valve regurgitation	Melody-Valve implantation in mitral position (8.5 months) Cardiac transplantation (10.5 months)	1.5 years	MOV, ARDS, sepsis
No. 9 (m)	Combined myectomy and membrane resection (2016)	1.3 years	Aortic valve stenosis, parachute mitral valve		3.5 years	Cardiorespiratory failure in metabolic imbalance, Suspected syndrome with developmental delay
No. 10 (f)	Combined myectomy and membrane resection (2001)	11.7 years	Shone’s with hypoplastic aortic annulus, mitral valve stenosis ASD closure (1994) De-Grouchy-Syndrome		17.4 years	Cardiac decompensation in palliative care

ARDS, acute respiratory distress syndrome; ASD, atrium septal defect; CoA, coarctation of the aorta; ECMO, extracorporeal membrane oxygenation, DORV, double outlet ventricle; HOCM, hypertrophic obstructive cardiomyopathy; TGA, transposition of the great arteries; MOV, multi organ failure; NEC, necrotizing enterocolitis; VSD, ventricle septal defect.

### Follow-up

#### Survival

**FIGURE 1:**
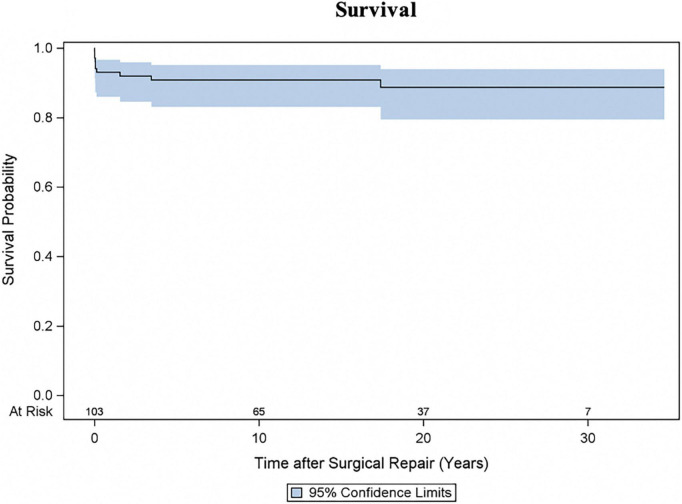
Survival following repair for subvalvular aortic stenosis (SAS). Kaplan-Meier estimated survival curve with 95% confidence interval (CI).

In addition to seven early deaths, three late deaths occurred, and Kaplan-Meier estimated survival was 90.8% (95% CI 83.0–95.1) at 10 years and 88.7% (95% CI 79.4–93.9) at 20 and 30 years (Figure 1). Two patients with HOCM underwent cardiac transplantation 10.5 months and 12.4 years after initial SAS repair respectively. One patient died 8 months after cardiac transplantation. Late deaths are seen in Table 3. At univariable Cox proportional hazards regression analyses (Table 4) age < 1 year at time of surgery (HR 6.4, 95% CI 1.9–22.2; *p* = 0.003), Shone’s complex (HR 4.4, 95% CI 1.1–16.9; *p* = 0.033), mitral stenosis (HR 7.0, 95% CI 2.0–24.8; *p* = 0.003) and concomitant mitral valve surgery (HR 4.7, 95% CI 1.2–18.3; *p* = 0.028) were statistically significantly associated with mortality.

**TABLE 4:** Risk factors for mortality (univariable Cox proportional hazards analyses).

	Univariable analysis		
Variable	Hazard ratio	Confidence	*P*-value
		interval (95%)	
Age^a^	0.9	0.7–1	0.065
Age < 1 year at time of surgery	6.4	1.9–22.2	**0.003**
Concomitant aortic valve involvement at time of surgery	1.1	0.2–5.1	0.911
Hypoplastic aortic arch	0.9	0.1–7.0	0.914
Shone’s complex	4.4	1.1–16.9	**0.033**
Mitral stenosis	7.0	2.0–24.8	**0.003**
Concomitant mitral valve surgery	4.7	1.2–18.3	**0.028**
Hypertrophic obstructive cardiomyopathy	1.3	0.3–6.1	0.751
Concomitant right ventricular outflow tract myectomy	1.4	0.2–10.8	0.766
Atrioventricular canal	0.8	0.1–6.0	0.792
Previous cardiac intervention (surgical and percutaneous)	0.8	0.2–2.7	0.657
Previous surgical intervention	0.9	0.3–3.2	0.877

^a^Continuous variable.

Statistically significant *P*-values are bold.

#### Reoperation

**FIGURE 2:**
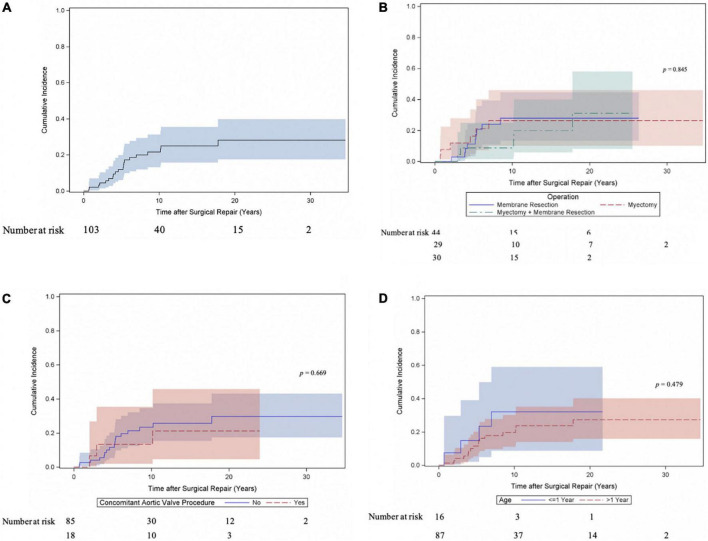
Cumulative incidence of reoperation for subvalvular aortic stenosis (SAS). **(A)** Cumulative incidence curve of reoperation for SAS. Curve with 95% confidence interval (CI). **(B)** Cumulative incidence of reoperation for SAS compared between patients following myectomy, membrane resection and combined myectomy, and membrane resection. Curves with 95% confidence interval (CI). **(C)** Cumulative incidence of reoperation for SAS compared between patients, who underwent concomitant aortic valve procedure at time of SAS repair and patients without valve involvement at SAS repair. Curves with 95% confidence interval (CI). **(D)** Cumulative incidence of reoperation for SAS compared between patients aged < 1 year at time of SAS repair and patients aged > 1 year at SAS repair. Curves with 95% confidence interval (CI).

**FIGURE 3:**
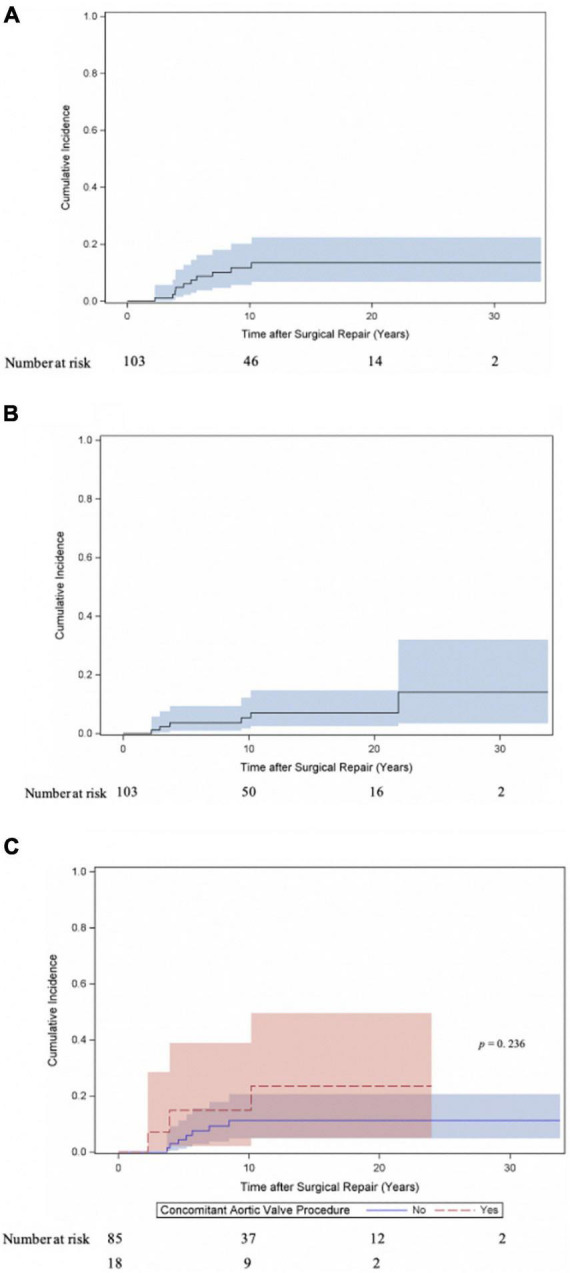
Cumulative incidence of reoperation on the aortic valve following repair for subvalvular aortic stenosis. **(A)** Cumulative incidence curve of any reoperation on the aortic valve (repair and replacement). Curve with 95% confidence interval (CI). **(B)** Cumulative incidence curve of aortic valve replacement. Curve with 95% confidence interval (CI). **(C)** Cumulative incidence of any reoperation on the aortic valve (repair and replacement) compared between patients, who underwent concomitant aortic valve procedure at time of SAS repair and patients without valve involvement at SAS repair. Curves with 95% confidence interval (CI).

Twenty patients underwent at least one reoperation for SAS. Five patients underwent a second reoperation for SAS and one patient a third reoperation. As shown in Figure 2A the cumulative incidence of reoperation for SAS was 21.6% (95% CI 13.2–31.4) at 10 years, 28.2% (95% CI 17.5–39.9) at 20 and 30 years. The incidence of reoperation for SAS did not differ (Gray test: *p* = 0.845) between the myectomy, membrane resection and combined myectomy, and membrane resection groups with 26.3% (95% CI 10.2–45.9), 27.9% (95% CI 13.5–44.4) and 31.2% (95% CI 8.3–58) at 20 years (Figure 2B). The incidence of reoperation for SAS did not differ (Gray test: *p* = 0.669) between patients, who underwent concomitant aortic valve procedures at SAS repair and patients without aortic valve procedures at SAS repair with 21.3% (95% CI 4.7–45.8) and 29.8% (95% CI 17.4–43.2) at 20 years respectively (Figure 2C). The incidence of reoperation for SAS did not differ (Gray test: *p* = 0.479) between patients aged < 1 year at SAS repair and patients aged > 1 year at SAS repair with 32.1% (95% CI 8.7–59) and 27.4% (95% CI 15.8–40.2) at 20 years respectively (Figure 2D). Also, at univariable Cox proportional cause-specific hazards regression analysis younger age at time of surgery was a risk factor for SAS reoperation (HR 0.9 for each increase in year; *p* = 0.015). Year of surgery did not correlate as factor for SAS reoperation (HR 1, 95% CI 0.9–1.1; *p* = 0.940). Ten patients underwent concomitant or solitaire reoperation on the aortic valve. The predominant indication for surgery on the aortic valve was valve regurgitation (aortic stenosis: 20%, 2/10; aortic regurgitation: 70%, 7/10; combined aortic valve disease: 10%, 1/10). The cumulative incidence of any reoperation on the aortic valve and aortic valve replacement were 13.5% (95% CI 6.8–22.5) and 7% (95% CI 2.5–14.7) at 20 years respectively (Figures 3A,B). The incidence of any reoperation on the aortic valve did not differ (Gray test: *p* = 0.236) between patients, who underwent concomitant aortic valve procedures at SAS repair and patients without aortic valve procedures at SAS repair with 23.5% (95% CI 5–49.5) and 11.3% (95% CI 4.9–20.6) at 20 years respectively (Figure 3C).

## Discussion

We reviewed our 30-years single center experience with pediatric SAS repair. Survival after SAS repair was good with a Kaplan-Meier estimated survival of 88.7% at 30 years. Reoperation rates in the setting of SAS recurrence were not to be neglected with a cumulative incidence of reoperation for SAS of 28.2% at 30 years. The surgical strategy concerning myectomy or membrane resection is based on the individual anatomy and the presence of a membrane and/or septal hypertrophy. The findings show that subvalvular aortic stenosis has a substantial recurrence rate, not only in patients with solitaire subaortic membrane, but also in patients with septal hypertrophy. The reoperation rates did not differ between the myectomy, membrane resection and combined myectomy, and membrane resection groups.

Cape et al. (7) propose that the development of SAS occurs due to subtle morphologic abnormalities, such as a steeper aortoseptal angle, which result in an altered septal shear stress, which triggers a genetic disposition leading to cell proliferation and structures in the LVOT. In patients with an atrioventricular canal defect, the goose neck-type of the LVOT contributes to an outflow tract stenosis due to the deficiency of the muscular septum and abnormal displacement of the mitral valve, which results in a long LVOT (2). In a systematic review and meta-analysis on pediatric SAS, Etnel et al. (4) observe that left ventricular outflow tract obstruction severity is correlated with progression of aortic valve regurgitation. Also, in natural history studies on pediatric SAS (8–12), a higher left ventricular outflow tract gradient at diagnosis has shown to be an independent predictor for aortic valve regurgitation, faster progression of aortic valve regurgitation and surgical intervention. Recurrence and reoperation rates remain a concern in pediatric patients with SAS with a recurrence rates ranging from 5 to 27% (9, 13–16). Complete removal of pathological membranous and fibromuscular subvalvular tissue from the LVOT is crucial, as residual tissue tends to form a recurrent obstruction. Donald et al. (3) found that extension of the membrane onto the aortic valve was a significant risk factor for SAS recurrence and reoperation.

Reported early mortality rates after SAS repair range from 0 to 4% (1, 3, 9, 14, 16, 17). The inclusion criteria regarding complexity of SAS (discrete and/or tunnel-like), indication for surgery and surgical strategy varied throughout the studies and need to be accounted for when comparing outcomes after SAS correction. We might have seen a higher early mortality with 6.8% (7/103) than reported in other studies as our cohort included also complex congenital heart disease patients undergoing SAS correction and all early deaths occurred in patients with complex heart disease or HOCM. Three patients out of the seven early deaths underwent additional cardiac surgery before death indicating the complexity of the underlying congenital heart disease. Additionally, ECMO support in the pediatric field was less evolved at the beginning of the study period. Four early deaths occurred in the setting of myocardial decompensation on ECMO support. Ruzmetov et al. (17), who included patients with discrete SAS (*n* = 140) and tunnel-like SAS (*n* = 50) reported an early mortality rate of 4% (7/190) and 10 late deaths. Actuarial survival including operative mortality of patients with discrete and tunnel-like SAS was 94 and 84% at 40 years (*p* = 0.14) respectively. In our cohort Kaplan-Meier estimated survival was 88.7% at 30 years. Etnel et al. (4) report a pooled early mortality of 2.05% (95% CI 0.61–6.41) and a pooled late mortality of 0.22%/year (95% CI 0.09–0.55). We found diagnosis of Shone’s complex, diagnosis of mitral stenosis, concomitant mitral valve surgery and age < 1 year at time of SAS correction to be predictive factors for mortality. Also, Serraf et al. (16) found tunnel-like SAS, concomitant mitral stenosis, coarctation of the aorta and hypoplastic aortic annulus to be risk factors for overall mortality. Permanent pacemaker implantation for complete AV-block was necessary in four patients undergoing solitaire myectomy (3.9%; 4/103) and was similar compared to other studies (9, 13, 15, 16) with rate of AV-block requiring pacemaker implantation from 3 to 6%.

Recurrence of SAS remains a concern in this pediatric cohort. Progression of aortic valve regurgitation might require valve intervention including aortic valve repair or aortic valve replacement. In their meta-analysis Etnel et al. (4) report a pooled reoperation rate of 2.04%/year (95% CI 1.52–2.62). In our cohort freedom from reoperation for SAS was 71.8% at 30 years. Ruzmetov et al. (17), who also included patients with discrete and tunnel-like SAS, report a reoperation rate of 2.7%/year. In our cohort the incidence of reoperation for SAS did not differ between the myectomy, membrane resection and combined myectomy, and membrane resection groups with 26.3, 27.9, and 31.2% at 20 years respectively. Also, the incidence of reoperation for SAS did not differ between patients, who underwent concomitant aortic valve procedures at SAS repair and patients without aortic valve procedures at SAS repair with 21.3 and 29.8% at 20 years, respectively. In the presented cohort the cumulative incidence of any reoperation on the aortic valve (aortic valve repair and replacement) and aortic valve replacement were 13.5 and 7% at 20 years respectively. Similar to other studies, the predominant indication for aortic valve surgery was aortic valve regurgitation (70%, 7/10). Donald et al. (3) found that patients with aortic valve involvement at initial SAS repair, whether peeling of SAS membrane from the aortic valve or other aortic valve repairs, required more aortic valve surgery in the follow-up period, compared to patients without aortic valve involvement at initial SAS repair.

### Study limitations

This study offers a long follow-up time with a median cardiac follow-up time of 10.5 years and near-complete mortality follow-up (94.2%). The study cohort is within the larger cohorts observed with this disease spectrum. This allows to assess the risk for reoperation over lifetime in the setting of SAS recurrence. As a typical limitation of a retrospective study design, it is possible that the variability regarding indication and an evolvement of perioperative management over the years are not fully accounted for.

## Conclusion

Surgical repair of subaortic stenosis in pediatric patients has good long-term outcomes, though recurrence rate of subaortic stenosis is not to be neglected and progression of aortic regurgitation might require aortic valve surgery. The incidence of reoperation for SAS did not differ between the myectomy, membrane resection and combined myectomy, and membrane resection groups. Patients who needed a combined membrane resection and septal myectomy are not more prone to recurrence than patients who underwent solitaire myectomy or membrane resection.

## Data availability statement

The original contributions presented in this study are included in the article/supplementary material, further inquiries can be directed to the corresponding author.

## Ethics statement

The studies involving human participants were reviewed and approved by Ethics Committee Medical University of Vienna, submission number: 1414/2019. Written informed consent from the participants or their legal guardian/next of kin was not required to participate in this study in accordance with the national legislation and the institutional requirements.

## Author contributions

JS: conceptualization, investigation, data curation, formal analysis, visualization, and writing—original draft. FW: investigation, data curation, and writing—review and editing. AK: conceptualization, formal analysis, visualization, and writing—review and editing. DW, HG, and SH: writing—review and editing. EB: validation and writing—review and editing. IM-B and GL: resources, validation, and writing—review and editing. DZ: supervision, resources, validation, and writing—review and editing. All authors contributed to the article and approved the submitted version.

## Abbreviations

CI: confidence interval
HOCM: hypertrophic obstructive cardiomyopathy
HR: hazard ratio
ICU: intensive care unit
IQR: interquartile range
LVOT: left ventricular outflow tract
SAS: subvalvular aortic stenosis.
